# Impact of Coexisting Uterine Adenomyosis on the Survival Outcome of Patients with Endometrial Cancer: A Retrospective Cohort Study

**DOI:** 10.31557/APJCP.2019.20.4.1185

**Published:** 2019

**Authors:** Sarana Boonlak, Apiwat Aue-Aungkul, Chumnan Kietpeerakool, Pilaiwan Kleebkaow, Bandit Chumworathayi, Sanguanchoke Luanratanakorn, Amornrat Temtanakitpaisan

**Affiliations:** *Department of Obstetrics and Gynecology, Faculty of Medicine, Khon Kaen University, Thailand. *

**Keywords:** Endometrial cancer, adenomyosis, risk factor, recurrence-free survival, overall survival

## Abstract

**Objective:**

To determine the effects of uterine adenomyosis on endometrial cancerrecurrence rates.

**Methods:**

This retrospective cohort study reviewed all consecutive patients diagnosed with endometrial cancerwho underwent total hysterectomy-based surgical staging at Srinagarind Hospital between January, 2010 and January, 2016. The patientswere divided into two groups:a uterine adenomyosisgroup and a non-adenomyosis group. Patient demographics, type of surgery, histopathology, stage of endometrial cancer, adjuvant treatment, and survival outcomes were compared.

**Results:**

A total 350 patients were enrolled, with 132 (37.71%) in the adenomyosis group and 218 (62.29%) in the non-adenomyosis group. Deep myometrial invasion and lymphovascular space invasion (LVSI) were more commonly found among patients who had no adenomyosis compared to those with adenomyosis(52.8% vs 39.4%, P=0.02 and 53.2% vs. 38.6%, P=0.01). There were no significant differences in terms of five-year recurrence-free survival (HR=1.47; 95%CI 0.88-2.44) and five-year overall survival (HR=0.81; 95%CI 0.43-1.53) between the two comparison groups.

**Conclusion:**

Coexisting uterine adenomyosis in endometrial cancer wasassociated withdeep myometrial invasion and LVSI but did not have significant impact on survival.

## Introduction

Endometrial cancer is the most common female genital tract malignancy in many developed countries (Birmann et al., 2016). In Thailand, endometrial cancer is the third most common gynecologic cancer, with an incidence of 2.8 per 100,000 per year (Wilailak and Lertchaipattanakul, 2016). The standard treatment for most patients with endometrial cancer is hysterectomy and bilateral salpingo-oophorectomywith or without surgical staging (Tangjitgamol et al., 2009).

The pathological factors that affect the reoccurrence rate of endometrial cancer include tumor size, myometrial invasion, presence of lympho-vascular space invasion (LVSI), tumor grade, and presence of tumors atthe lower uterine segment (Morice et al., 2016; Kong et al., 2017; Bishop et al., 2017; Güngördük et al., 2018). The choice of adjuvant treatment for women with early-stage endometrial cancer is, therefore, based on these factors (Koh, 2018).

Uterine adenomyosis is characterized by the presence of endometrial stroma and glands within the myometrium at least one low-power field from the basis of the endometrium. It is a benign condition and commonly found in women of reproductive age. The etiologies of adenomyosisareunclear but there is some evidence that local hyperestrogenismin the uterus, such as endometrial hyperplasia, uterine leiomyoma, and endometrial cancer, may increase the riskof adenomyosis (Benagiano, 2012).

Previous studies have foundadenomyosisin 40-70% of endometrial cancerspecimens (Ismiil et al., 2007; Taneichi et al., 2014). However, these studies have beeninconclusive regarding theeffect of adenomyosis on progression, recurrence,and survival ratesin casesendometrial cancer. Several studies found that endometrial cancer arising from adenomyosis was associated with deep myometrial invasion and poor survival outcomes (Ismiil et al., 2007; Ismiil et al., 2007; Taneichi et al., 2014; Machida et al., 2017). However, numerous studies have also shownthat adenomyosis is associated with lower risks of LVSI, myometrial invasion, and lymph node involvement (Matsuo et al., 2014; Torre et al., 2015; Gizzo et al., 2016).

The inconclusive results from the previous studies based on the present or absent of adenomyosis in endometrial cancer patients. So the aim of this study was to evaluate the survival outcomes of coexisting endometrial cancer anduterine adenomyosis.

## Materials and Methods

*Study design*

This study was approved by the institutional ethical review board. Inclusion criteria included all patients with diagnosed endometrial cancer who underwent surgical staging at Srinagarind Hospital between January 1, 2010 and January 1, 2016. Exclusion criteria weremetastatic cancer to the endometrium, synchronous tumors, and patient having received neoadjuvant therapy. 

Patient age, parity, body mass index (BMI), tumor stage, date of surgery, type of surgical staging, and adjuvant therapy were collected from medical records. Histopathological slides were also reviewed and interpreted by agynecological pathologist (Kleebkaow P). Eachuterine specimen (excluding the isthmus and cervix)was dividedamong eight to 12 slides for examination. Diffused adenomyosis was defined as the presence of adenomyosisinmore than half of the uterine slides. Focal adenomyosis was defined as the presence of adenomyosisin fewer than half of the uterine slides. We used a dualistic classification of endometrial cancers according to Bokhman subtype (Suarez et al., 2017). Type I includedendometrioid adenocarcinoma grades 1 and 2. Type IIconsistedendometrioid adenocarcinoma grade 3, clear cell carcinoma, and serous carcinoma. Other factors that can affect the recurrence rate of endometrial cancerwere also evaluated such as tumor grade, depth of myometrium invasion, cervical stromal invasion, presence of lympho-vascular space invasion (LVSI), adnexa involvement, lymph node status, presence of uterine leiomyoma or endometriosis, and tumor stage according to the International Federation of Gynecology and Obstetrics (FIGO) 2009 classification (Sorosky, 2012).

Adjuvant treatment, such as vaginal brachytherapy, whole pelvic radiation, chemotherapy, or combined therapy, were administeredaccording to NCCN guidelinesdepending on the histopathologicalfindings from surgical specimen.

Aftertreatment was completed, all patients were followed up on every three months for the first 24 months and every six months forthe next five years in order to evaluate thelong-term outcomes. At each follow-up visit, data regarding the patient’smedical history was obtained and physical and pelvic examinations were performed.Survival analysis was based on the Kaplan-Meier method and results were compared using the log-rank test.

Recurrence-free survival (RFS) was defined as the time from the date of primary surgery to the detection of recurrence or the latestfollow-up. Overall survival (OS) was defined as the time from the date of primary surgery to death or the time that the patient was still alive at the time of the collection from the last data search from the Thai Civil Registration.

The χ^2^ test and student’s t-test for unpaired data were used for comparing between the groups. For predictors with a p-value of less than 0.20 in univariate analysis (log-rank test), Cox proportional-hazards regression was used to determine the independent impact of coexistingadenomyosis onsurvival outcomes. Collinearities between the factors included in the multivariate analyses were checked. All statistical analyses were performed using the SPSS version 17 (SPSS Inc., Chicago, Ill., USA). A p-value < 0.05 was considered to indicate statistical significance.

The primary end point for the cohort study was the recurrence rate between adenomyosis and non-adenomyosis groups. Previous study from Sorbe et al., (2014) found 30% of endometrial cancer patients had experienced recurrent disease. We needed 134 participants in each group to detect a 50% difference (15% of recurrent disease in endometrial cancer patients with adenomyosis) with 80% power using a two-sided significance level of .05. So we needed to recruit 134 subjects per arm, for a total of 268 subjects.

## Results

A total of 350 patients withendometrial cancer who underwent surgical staging between January 1, 2010 and January 1, 2016 in our institution were identified. The median follow-up time was 39.5 months (interquartile range or IQR 20-67 months). The mean patient age was 58.1 years (range 31-84 years), anduterine adenomyosis was found about 37.7% (95%CI 32.6-42.8%) of the patients enrolled. Focal adenomyosiswas the most common type found (64.4%). Diffuse adenomyosis was diagnosed in only 47(35.6%) specimens.Most of the patients were under 60 years old (62%), multiparous (74.2%), and had a BMI of less than 30 kg/m^2^ (87.7%). 

Almost all of the patients presented when the disease was in its early stages. The clinical characteristics of patients diagnosed endometrial cancer – both with (n=132) and without (n=218) uterine adenomyosis –arelisted in Table 1. The median age at diagnosis in the uterine adnomyosis group was 59 years (range 36 to 80 years), and the most common histologic type of endometrial cancer was type I (65.2%). As of January 31, 2018, the median duration of follow-up from the date of surgery was 38.5 months (range:one to 98 months), the recurrence rate was15.9% and the death-rate was10.6%.

**Table 1:** Patient Characteristics of Participants

Characteristics	Adenomyosis (n =132)	No adenomyosis (n=218)	P-value†
Age at diagnosis			
Median	59	58	-
Range	36.0-80.0	31.0-84.0	
Follow-up time from surgery date			
Median	38.5	41	-
Range	1.0-98.0	5.0-98.0	
Parity			
Nulliparity	33 (25.0)	56 (25.7)	0.89
Multiparity	99 (75.0)	162 (74.3)	
BMI			
Mean ± SD	25.4 ± 4.8	25.2 ± 4.2	0.39
Presence of myoma uteri			
Yes	58 (43.9)	68 (31.2)	0.02
No	74 (56.1)	150 (68.8)	
Lymphadenectomy			
Yes	108 (81.8)	176 (80.7)	0.8
No	24 (18.2)	42 (19.3)	
Adjuvant treatment			
Yes (vaginal brachytherapy, whole pelvic radiation, chemotherapy, or combined modality)	76 (57.6)	155 (71.1)	0.01
No	56 (42.4)	63 (28.9)	
Treatment outcome			
Recurrence	21 (15.9)	51 (23.4)	-
Death	14 (10.6)	29 (13.3)	-

Data are presented as number (percentage) unless stated otherwise; BMI, body mass index; †, Pearson Chi-square, two-sided, P-value<0.05

**Table 2:** Tumor Characteristics of Participants

Characteristics	Adenomyosis (n =132)	No adenomyosis (n=218)	P-value†
Histology, No (%)			
Type I	86 (65.2)	151 (69.3)	0.42
Type II	46 (34.8)	67 (30.7)	
FIGO Staging			
I	84 (63.7)	127 (58.3)	0.3
II	14 (10.6)	20 (9.2)	
III	29 (21.9)	52 (23.8)	
IV	5 (3.8)	19 (8.7)	
Myometrial invasion			
Inner half	80 (60.6)	103 (47.2)	0.02
Half or outer	52 (39.4)	115 (52.8)	
Cervical stromal involvement			
Yes	29 (22.0)	62 (28.4)	0.18
No	103 (78.0)	156 (71.6)	
Presence of LVSI			
Yes	51 (38.6)	116 (53.2)	0.01
No	81 (61.4)	102 (46.8)	
Nodal metastasis			
Yes	21 (15.9)	37 (17.0)	0.97
No	88 (66.7)	144 (66.0)	
Unknown	23 (17.4)	37 (17.0)	
Endometriosis			
Yes	15 (11.4)	13 (6.0)	0.07
No	117 (88.6)	205 (94.0)	

Data are presented as number (percentage) unless stated otherwise; LVSI, lymphovascular space invasion; FIGO, the International Federation of Gynecology and Obstetrics; †, Pearson Chi-square, two-sided, P-value<0.05

**Table 3:** Risk Factors for Recurrence-Free Survival in Endometrial Cancer According to Cox-Proportional Hazard Regression

Factors	Number at risk	5-year survival proportion (%)	Univariate		Multivariate	
			HR (95%CI)	P-value	HR (95%CI )	P-value
Adenomyosis						
Yes	21/132	82.1	0.68 (0.41-1.13)	0.14	1.43 (0.85-2.41)	0.16
No	51/218	74.4	Reference		Reference	
Stage						
III-IV	52/105	43.9	8.77(5.22-14.76)	< 0.01	7.13 (3.98-12.77)	<0.01
I-II	20/245	90.8	Reference		Reference	
Histology						
Type II	39/113	58.1	3.14 (1.97-5.01)	< 0.01	1.76 (1.07-2.89)	0.02
Type I	33/237	85.3	Reference		Reference	
Age						
Over 60 years	34/133	71.3	1.62 (1.02-2.58)	0.04	1.63 (1.01-2.63)	0.04
60 years or younger	38/217	81.1	Reference		Reference	
LVSI						
Yes	51/167	64.2	3.16 (1.89-5.26)	< 0.01	1.14 (0.65-2.01)	0.66
No	21/183	88.5	Reference			
BMI						
Less than 30	64/307	89.5	1.09 (0.52-2.27)	0.83	Variable removed	
30 or more	8/43	76.2	Reference			
Myoma uteri						
Yes	26/126	79.8	0.98 (0.61-1.58)	0.93	Variable removed	
No	46/224	75.8	Reference			

HR, hazard ratio; LVSI, lymphovascular space invasion; BMI, body mass index.

**Figure 1:**
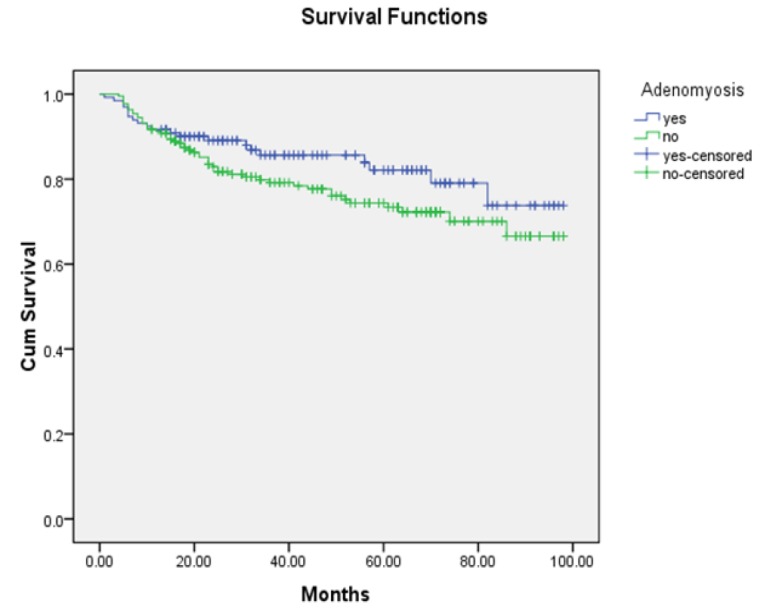
Recurrence-Free Survival Curves of Tendometrial Cancer Patients with and without Adenomyosis(P=0.13)

**Figure 2:**
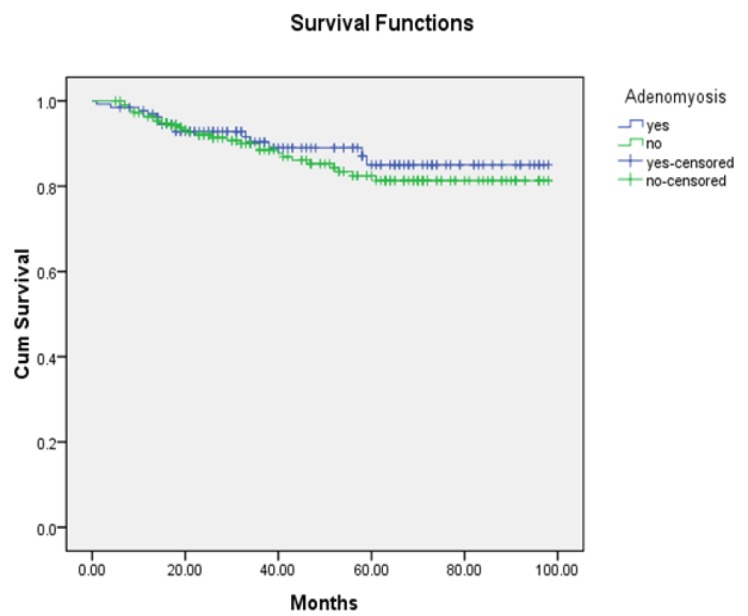
Overall Survival Curves of Endometrial Cancer Patients with and without Adenomyosis(P=0.51)

Patients with coexisting uterine adenomyosis were significantly more likely to have myoma uteri and less likely to need adjuvant therapy after surgery than those without (43.9% vs 31.2% and 57.6% vs 71.1%, respectively). The treatment after surgical stagingincluded no adjuvant therapy (119, 34.0%), vaginal brachytherapy (9, 2.6%), whole pelvic radiation (14, 4.0%), systemic chemotherapy (51, 14.6%), and combined chemo-radiation (157, 44.9%).

In terms oftumor characteristics, there were significant differences observed between the two groups in the rates of myometrial invasion in the outer half of myometrium (39.4% vs 52.8%) and LVSI (38.6% vs 53.2%). However, therewas no significant difference observed with regard to lymph node metastasis. In subgroup analysis, type of adenomyosis (focal vs diffuse) was not significantly associated with deep myometrial invasion (38.8% vs 40.4%) or advanced disease (25.9% vs 25.5%).

There were no significant differences in terms of five-year recurrence-free survival oroverall survival between patients with coexisting adenomyosis and those without (82.1% vs 74.4% and 85.0% vs 82.4%, respectively). According to univariate analysis, endometrial cancer coexisting with adenomyosis was not associated with five-year recurrence-free survival (HR=1.47; 95%CI 0.88-2.44; p=0.14)orfive-year overall survival (HR=0.81; 95%CI 0.43-1.53; p=0.51) when compared with endometrial cancer alone, as shown in Figures 1 and 2. According tosubgroup analysis, the type of uterine adenomyosis did not affect recurrence-free (p=0.30) or overall survival (p=0.77).

The significant prognostic factors for recurrence-free survival were age over 60 years, histological typeII, stage III-IV, and presence of LVSI. Nevertheless, BMI and presence of uterine myoma were not significantly associated with recurrence-free survival in patients with endometrial cancer.

According tomultivariate analysis, the presence of uterine adenomyosis in endometrial cancer was not associated with recurrence-free survival (HR=1.43; 95%CI 0.85-2.41) when adjusted for age, histology of the tumor, presence of LVSI, and stage (Table 3).

## Discussion

Previous studies have found the rate of coexisting adenomyosis in endometrial cancer to be approximately 40-70% (Ismiil et al., 2007; Taneichi et al., 2014). In the present study, we found coexisting adenomyosis in 37.71% of hysterectomy specimens of endometrial cancer. Deep myometrial invasion and LVSI were more commonly found among patients without adenomyosis than in those with adenomyosis (52.8% vs 39.4% and 53.2% vs. 38.6%, respectively). 

A study by Matsuo et al., (2014) found that adenomyosis significantly affected tumor progression and survival outcomesin cases of endometrial cancer. Moreover, they found that adenomyosis was associated with reduced myometrial invasion. Possible explanations for these associationsare that the adenomyosis increased the levels of some cytokines that exert anti-tumor effects, thickening of the endometrial stroma due to secretion of estrogen, or that the inflammatory cytokines in adenomyosis caused a mechanical block against the invasion of endometrial cancer to the myometrium.Erkilinç et al., (2018)reported that patients with endometrial cancer and no adenomyosis experienced higher rates of myometrial invasion and lymphovascular space invasion, had larger tumor diameters, and underwent a greater number of adjuvant treatments than those with adenomyosis. Gizzo et al., (2016) evaluated prognosis estimation of coexistence of adenomyosis in endometrioid endometrial cancer and found that the coexistence of adenomyosis and endometrioid endometrial cancer was associated with myometrial invasion, LVSI, lymph node involvement, and tumor size. Our study reveals that adenomyosis could reduce myometrial invasion, risk of lympho-vascular space invasion, and the amount of lower adjuvant treatment required. 

The present study found that survival outcomes (recurrence rate and overall survival rate) were higher in the adenomyosis group but not to a statistically significant extent. This result is consistent with those of some previous studies, which found that the presence of adenomyosis had no independent effect on survival outcomes (Musa et al., 2012; Taneichi et al., 2014). However, there are other studies that have found contradictory results. A study by Matsuo et al., (2014), for example,found that thepresence of adenomyosis decreased the risk of disease recurrence after surgery (HR = 0.53; 95 % CI, 0.30–0.92). Moreover, Erkilinç et al., (2018) reported that the presence of adenomyosis was associated with higher overall survival (HR = 0.20; 95% CI, 0.03–0.68). A possible explanation for these results is that most participants in our study received adjuvant treatment, which can reduce tumor recurrence (Eggink et al., 2017; Iwase et al., 2018; Qu et al., 2018). In this study, patients received adjuvant treatment about 66% of all patients. When compared with previous studied, we found only half or less patients received adjuvant treatment (Eggink et al., 2017; Qu et al., 2018).

A study by Erkilinç et al., (2018) found no significant association between the location of adenomyosis and depth of tumor invasion (p=0.19). In this study, the most common type of uterine adenomyosis was focal adenomyosis or adenomyoma (64.4%) and there was no association between the type of adenomyosis and depth of myometrial invasion, stage of disease, orsurvival outcomes. 

Some previous studies have described an association between tumor histology and survival outcomes (Ueda et al., 2010; Huijgens and Mertens, 2013; Ouldamer et al., 2016). Huijgens and Mertens (2013), for example, found thattype II tumors were significantlyassociated with disease recurrence (HR = 3.76; 95%CI, 1.73-8.18). Additionally, Ueda et al., (2010) reported that non-endometrioid tumors were significantly associated with disease recurrence (HR = 2.77; 95%CI, 1.22-6.30). Furthermore, Ouldamer et al., (2016) found tumor recurrence to be independently associated with type II endometrial cancer (HR =2.67; 95%CI, 1.29-5.50). This is consistent with the results ofour study, which also found type II cancer to be associated with increased the risk of disease recurrence (HR = 1.76, 95% CI 1.07-2.89).

In our study, patients over 60 years old carried a higher risk of recurrence (HR = 1.63; 95%CI, 1.01-2.63), which contradicts the results of some previous studies (Ueda et al., 2010; Ouldamer et al., 2016). Ueda et al., (2010), for example, foundthat patient age was not a significant predictor of cancer recurrence (HR = 0.79; 95%CI, 0.35-1.83). In addition, Ouldamer et al., (2016) found that age over 60 years was not significantly associated with recurrence-free survival (HR = 1.69; 95%CI, 0.87-3.30).

Previous studies have consistently reported an association between cancer stage and survival outcome (Huijgens and Mertens, 2013; Ouldamer et al., 2016).Huijgens and Mertens (2013) reported that the recurrence rate was significantly higher in patients with FIGO stage III-IV than in those with FIGO stage I-II (p<0.001). In addition,Ouldamer et al., (2016) reported that FIGO stage III endometrial cancer was a significant predictor for disease recurrence (HR = 2.00; 95%CI, 1.11-3.60). 

Our study also found FIGO stage III-IV to beassociated with disease recurrence (HR = 7.13; 95% CI,3.98-12.77).

The strength of the current study was that it was able to determine the impact of different types of adenomyosis on survival outcome in patients with endometrial cancer. In addition, all surgical specimens were reviewed by an experienced pathologist. However, as it was based on retrospective data collection, some data were unavailable such as the location of adenomyosis and tumor diameter. In conclusions, the presence of adenomyosis in endometrial cancer had no significant impact on survival outcomes.

## Funding Statement

This study was supported by a grant from the KhonKaen University Faculty of Medicine (Thailand; Grant Number IN60232).

## Statement conflict of Interest

No conflict of interest.
